# *Streptomonospora litoralis sp*. nov., a halophilic thiopeptides producer isolated from sand collected at Cuxhaven beach

**DOI:** 10.1007/s10482-021-01609-4

**Published:** 2021-08-06

**Authors:** Shadi Khodamoradi, Richard L. Hahnke, Yvonne Mast, Peter Schumann, Peter Kämpfer, Michael Steinert, Christian Rückert, Frank Surup, Manfred Rohde, Joachim Wink

**Affiliations:** 1 Helmholtz Centre for Infection Research (HZI), Microbial Strain Collection (MISG), 38124 Braunschweig, Germany; 2 Leibniz Institut DSMZ-German Collection of Microorganisms and Cell Cultures, 38124 Braunschweig, Germany; 3 Institut Für Angewandte Mikrobiologie, Justus-Liebig-Universität Giessen, Heinrich-Buff-Ring 26–32, 35392 Giessen, Germany; 4 Institut Für Mikrobiologie, Technische Universität Braunschweig, Spielmannstrasse. 7, 38106 Braunschweig, Germany; 5 Technology Platform Genomics, Center for Biotechnology, Bielefeld University, 33615 Bielefeld, Germany; 6 Microbial Drugs Department, Helmholtz-Centre for Infection Research, 38124 Braunschweig, Germany; 7 Central Facility for Microscopy, Helmholtz Centre for Infection Research, 38124 Braunschweig, Germany

**Keywords:** Actinobacteria, Polyphasic taxonomy, Rare actinomycete, *Streptomonospora litoralis* sp., Thiopeptide

## Abstract

Strain M2^T^ was isolated from the beach of Cuxhaven, Wadden Sea, Germany, in course of a program to attain new producers of bioactive natural products. Strain M2^T^ produces litoralimycin and sulfomycin-type thiopeptides. Bioinformatic analysis revealed a potential biosynthetic gene cluster encoding for the M2^T^ thiopeptides. The strain is Gram-stain-positive, rod shaped, non-motile, spore forming, showing a yellow colony color and forms extensively branched substrate mycelium and aerial hyphae. Inferred from the 16S rRNA gene phylogeny strain M2^T^ affiliates with the genus *Streptomonospora.* It shows 96.6% 16S rRNA gene sequence similarity to the type species *Streptomonospora salina* DSM 44593^ T^ and forms a distinct branch with *Streptomonospora sediminis* DSM 45723^ T^ with 97.0% 16S rRNA gene sequence similarity. Genome-based phylogenetic analysis revealed that M2^T^ is closely related to *Streptomonospora alba* YIM 90003^ T^ with a digital DNA-DNA hybridisation (dDDH) value of 26.6%. The predominant menaquinones of M2^T^ are MK-10(H_6_), MK-10(H_8_), and MK-11(H_6_) (> 10%). Major cellular fatty acids are iso-C_16:0,_ anteiso C_17:0_ and C_18:0_ 10-methyl. The polar lipid profile consisted of diphosphatidylglycerol phosphatidyl glycerol, phosphatidylinositol, phosphatidylcholine, phosphatidylethanolamine, three glycolipids, two unknown phospholipids, and two unknown lipids. The genome size of type strain M2^T^ is 5,878,427 bp with 72.1 mol % G + C content. Based on the results obtained from phylogenetic and chemotaxonomic studies, strain M2^T^ (= DSM 106425^ T^ = NCCB 100650^ T^) is considered to represent a novel species within the genus *Streptomonospora* for which the name *Streptomonospora litoralis* sp. nov. is proposed.

**Supplementary Information:**

The online version contains supplementary material available at 10.1007/s10482-021-01609-4.

## Introduction

Despite the increasing number of infectious diseases caused by drug resistant bacteria, the development of new antibiotics by the pharmaceutical industry has largely been abandoned because it is challenging and not lucrative (World Health Organization 2017). Actinobacteria (Stackebrandt et al. 1997), especially representatives of the genus *Streptomyces* Waksman and Henrici 1943 (Skerman et al. 1980) are major producers of bioactive substances as they are responsible for the production of more than 70% of all medical important antibiotics. Due to the rediscovery of many known compounds from *Streptomyces* species in the past years, researchers have focused more on rare Actinobacteria (non-*Streptomyces*) as source to discover new antibiotics (Sivalingam et al. 2019). It has become clear that the identification of new antimicrobial compounds is strongly related with the discovery of novel species (Thumar et al. 2010). Therefore, mining of microorganisms from unexploited or underexplored habitats is considered a promising approach to discover novel antibiotics (Baumann et al. 2014). Soil is the most prominent and rich habitat for rare Actinobacteria. In comparison with soil, other habitats such as water, ocean sediments, or beach sands can be regarded as neglected in terms of rare Actinobacteria isolation approaches. The ability of rare Actinobacteria to adapt to many different habitats opens up the possibility of finding new species from the untapped environments (Lam 2006). The number of rare Actinobacteria which has been isolated from marine habitats such as sands or sediments is very low (Subramani et al. 2019). It is therefore worthwhile to search in such little-studied habitats for novel rare Actinobacteria, which may be potent producers of new bioactive natural compounds. The genus of *Streptomonospora* corrig. Cui et al. (2001) emended by Li et al. (2003) was first proposed by description of the type species *Streptomonospora salina* YIM 90002^ T^*,* which belongs to the family Nocardiopsacea (Rainey et al. 1996) emended by Zhi et al. (2009), in the phylum Actinobacteria (Thrash and Coates 2010). Members of the genus *Streptomonospora* are aerobic, Gram-positive and grow with branching hyphae. Substrate mycelium is not fragmented. Spore shapes in chains are rod-oval, surfaces are wrinkled, whereas single spores are oval to round and can exhibit a smooth or wrinkled surface (Zhang et al. 2013). In the original description of the type species *S. salina* (Cui et al. 2001), Cui distinguishes between spore chains on the aerial mycelium and single spores formed on substrate mycelium. These two different spore types on aerial and substrate mycelium were described for 8 of 11 till now described species: *S. salina*, *S. alba* (Li et al. 2003), *S. algeriensis* (Meklat et al. 2014), *S. amylolytica* and *S. flavalba* (Cai et al. 2009), *S. halotolerans* (Zhao et al. 2015), *S. tuzyakensis* (Tatar et al. 2016) and *S. sediminis* (Zhang et al. 2013). The three species *S. nanhaiensis* (Zhang et al. 2013), *S. arabica* (Hozzein and Goodfellow, 2008) and *S. halophila* (Cai et al. 2008) formed both single spores and spore chains on aerial mycelium.

The genus *Streptomonospora* comprises halophilic strains, which have been isolated mostly from salty habitats, such as salty lakes, hipper saline soils, seas or oceans (Li et al. 2003; Cai et al. 2008, 2009).

In frame of an investigation of the secondary metabolite production capacity of rare Actinobacteria obtained from beach sands, strain M2^T^ was isolated from a sand sample collected at Cuxhaven, North Sea, Germany (53° 52′ 39.5″ N, 8° 41′ 22.4″ E) on 20^th^ of July 2017. In our preceding study, we showed that M2^T^ produces the two new thiopeptides litoralimycin 1 (A) and 2 (B) with cytotoxic activity (Khodamoradi et al. 2020). Here, we report on the description of new *Streptomonospora* species with the type strain M2. Furthermore, we report on the identification of sulfomycins 3–5 as further thiopeptide secondary metabolites from M2^T^, as well as the assignment of a putative biosynthetic gene cluster encoding the biosynthesis of the M2^T^ thiopeptide compounds.

## Material and methods

### Isolation and culture conditions

Strains M2^T^ and M3 were isolated from a sand sample of beach of Cuxhaven, Wadden Sea, Germany. The sand sample was suspended in sterile water and serially diluted. The 1/1000 diluted sample was cultivated on agar containing soluble starch 10 g/l, casein 1 g/l, K_2_HPO_4_ 0.5 g/l, MgSO_4*_7H_2_O 5 g/l, agar 20 g/l in 1000 ml deionized water at pH 7.3, supplemented with 35.5 g/l sea water (Coral Ocean). The plates were incubated at 30 °C and checked for growth after 1–2 weeks. Sub-culturing was performed for purification on GYM medium (glucose 4 g/l, yeast extract 4 g/l, malt extract 10 g/l, CaCO_3_ 2 g/l, agar 12 g/l, sea water 35 g/l in 1000 ml deionized water at pH 7.2) according to Wink et al. (2002).

### Molecular analysis

Genomic DNA was isolated from a five-day old GYM liquid culture by using Invisorb Spin Plant Mini Kit 250 (Stratec). The 16S rRNA gene sequence was amplified by PCR. For gene amplification the primers F27 (5’AGAGTTTGATCCTGGCTCAG-3’) and R1541 (5^’^-AAGGAGGTGATCCAACCGCA-3’) were used. DNA amplification was performed by PCR with the following protocol: 5 min at 95 °C; 34 cycles of 30 s at 94 °C, 30 s at 52 °C and 2 min at 72 °C, plus an additional 10 min cycle at 72 °C. The PCR product was analysed by agarose gel electrophoresis. The PCR product was purified with the Nucleo Spin Microbial DNA Mini kit (MACHEREY–NAGEL). The almost full-length 16S rRNA gene fragment (1501 bp) was sequenced by using the 96-capillary system from Applied Biosystems (ABI), 3730xl DNA Analyser (Weisburg et al. 1991). The 16S rRNA gene sequence was compared with sequences from the NCBI database by BLAST analysis (http://www.ncbi.nlm.nih.gov/BLAST) (Pruitt et al. 2005), as well as with sequences available at the EZTaxon database (www.ezbiocloud.net/) (Yoon et al. 2017). The phylogenetic tree based on 16S rRNA gene sequences was created by using the ARB release 5.2 program. Using the “All-Species Living Tree" Project (LTP) database (release LTPs128). All sequences not included in the LTP database were aligned according to the SILVA seed alignment using the SILVA Incremental Aligner (SINA) version v1.2.9, before the sequences were implemented into the ARB database. The alignment of all sequences of *Streptomonospora* type strains was manually corrected including secondary structure information. DNA-DNA hybridisation (DDH) experiments were performed between strain M2^T^ and the three closest type strains *Streptomonospora halophila* DSM 45075^ T^, *Streptomonospora arabica* DSM 45083^ T^, and *Streptomonospora sediminis* DSM 45723^ T^ according to the method of Ziemke et al. (1998).

### Genome sequencing, annotation and analysis

Genomic DNA for whole genome sequencing was extracted with the procedure described by Kieser et al. (2000). Genomic DNA was used to construct two sequencing libraries, one using the Rapid Barcoding Kit run on a R9.4.1 flow cell Oxford Nanopore Technologies, Oxford, UK (ONT), and one using the TruSeq PCR-free Library Prep Kit run on a MiSeq sequencing platform (lllumina, Eindhoven, NL). The first sequencing approach resulted in the acquisition of 26,561 reads containing, 337.1 Mbp. The second delivered 3,864,942 reads containing 1077.9 Mbp. The ONT data were assembled using canu v.1.7 (Koren et al. 2017), resulting in a single contig of 5,878,427 bp. After trimming of the overlapping ends and rotating the genome based on *dnaA*, the resulting contig was first polished using minimap2 v.2.10 (Li 2018) and nanopolish v.0.11.0 using the ONT raw fast5 data (Loman et al. 2015). The polished contig was then subjected to 10 rounds of additional polishing using pilon v.1.22 and the Illumina data mapped with either bwa mem v.0.7.12 (Li 2018) (round 1–5) or bowtie2 v.2.3.3 (Langmead and Salzberg 2012) (round 6–10). In all cases, samtools v.1.9 (Li 2018), was used for handling mapping data. In parallel, the Illumina data were assembled using the newbler v.2.8 assembler (454 Life Sciences, Branford, CT, USA), resulting in an assembly consisting of 89 contigs in 26 scaffolds. Both assemblies were combined using Consed v27.0 (Gordon and Green 2013), with the Illumina contigs being used for manual correction of the remaining errors in repetitive regions in the polished canu assembly. The manual inspection resulted in the identification of a second, circular “replicon”: a part of the chromosome consists of a prophage that is also present as an episome at low abundance (plasmid phiM2). Both contigs were annotated using Prokka v.1.11 (Seemann 2014). The annotated replicons have been deposited at DDBJ/ENA/GenBank under the accessions CP036455 (chromosome) and CP036456 (plasmid phiM2). The whole genome sequence of strain M2^T^ is released in IMGM, (https://img.jgi.doe.gov/) with the IMG genome ID 2,845,838,682. Whole-genome phylogeny was generated using the TYGS server (https://tygs.dsmz.de) (Seemann 2014). The digital DNA-DNA hybridisation (dDDH) values between the genomes of the strains were calculated using the Genome-to-Genome Distance Calculator GGDC 2.1 (http://ggdc.dsmz.de) (Meier-Kolthoff et al. 2013). Bioinformatic analysis for the identification of potential secondary metabolite gene clusters has been performed with the webtool antiSMASH 5.0 (https://antismash.secondarymetabolites.org) (Blin et al. 2019).

### MALDI-TOF analysis and ribotyping

Preparation of samples for matrix-assisted laser-desorption/ionisation time-of-flight spectrometry (MALDI-TOF MS) from approximately eight mg biomass (cultivated in liquid GYM medium, 30 °C for 5 days) and recording of spectra were performed according to the protocol of Schumann and Maier (2014) as described in detail by Wink et al. (2017). A dendrogram based on the similarity of MALDI-TOF mass spectra was generated by the BioTyper software (version 4.1.80, Bruker Daltonics*)*. Automated ribotyping of the isolates M2^T^ and *Streptomonospora halophila* DSM 45075^ T^ was accomplished according to Schumann and Pukall (2017), by using the RiboPrinter system (Hygiena) and *Pvu*II as restriction enzyme. A dendrogram based on pattern similarity was created using the software package BioNumerics, Applied Maths (Sint-Martens-Latem, Belgium).

### Morphological and physiological analysis

Culture characteristics were investigated after 14 days of incubation at 30 °C, on several ISP (International *Streptomyces* Projects) media ISP2-ISP7 (Shirling and Gottlieb 1966). The two synthetic media from Suter (1978), with tyrosine and without tyrosine were used for detection of melanin pigment production (Suter 1978). All media were supplemented with 7% NaCl (w/v). For long-term storage, a section of the agar containing bacterial colonies and aerial mycelium were stored in glycerol 20% at −80 °C (Wink 2002). Colors of substrate mycelium, aerial mycelium, and diffusible pigments were identified by comparison with the RAL color card (Reichsausschuß für Lieferbedingungen—Deutsches Institut für Gütesicherung e.V.) (Kelly 1964).

The utilisation of carbohydrates was followed employing microtiter plates (Williams 1989), with ISP9 medium (International *Streptomyces* Project) as described by Shirling and Gottlieb (1966). Growth in medium with different NaCl concentrations was investigated on basal medium with 0, 2.5, 5, 7, 10, 15, 20 and 30% NaCl (w/v) (Pridham and Gottlieb 1948). Growth at different temperatures 4, 16, 20, 25, 30, 37, 45 and 55 °C was determined on GYM medium for up to 14 days (Kutzner 1981). The pH tolerance was determined in liquid ISP2 medium buffered with HIPS (Carl Roth GmbH + Co.KG) at 30 °C for 10 days, according to Kutzner (1981). Enzyme utilisation was investigated in triplicates by API Coryne (bioMérieux Ref. 20,900) and API ZYM (bioMérieux Ref. 25,200).

The decomposition of polysaccharides and peptides was investigated in 96-well plates, following the protocol of Panschin et al. (2016). Each strain was tested in triplicates with respective controls and was incubated at 25 °C for up to 14 days.

To analyse the structure of the mycelium and spores, the cultures were grown on a complex solid growth medium (DSMZ medium 65: glucose 4.0 g/l, yeast extract 4.0 g/l, malt extract 10.0 g/l, CaCO_3_ 2.0 g/l, agar 15,0 g/l; pH 7.2). A section of the agar comprising a bacterial lawn was fixed in 5% glutaraldehyde according to the description of Wink et al. (2002)*.* Samples were critical-point-dried and gold–palladium-sputtered and the morphology of the spores was detected using a Zeiss Merlin field emission scanning electron microscopy (FESEM) with an Everhart–Thornley SE-detector and an Inlens-SEM detector in a 25:75% ratio utilising the SEM Smart software version 5.05To.

### Chemotaxonomic characterisation

To analyse the whole-cell diaminopimelic acid isomers and sugars, samples were prepared as described by Hasegawa et al. (1983). Polar lipids were identified with thin layer chromatography (TLC) plates by development in two directions. First direction was run with solvent A (chloroform–methanol-water 65:25:4). The TLC plate was dried at room temperature before it was developed in the second direction in solvent B (chloroform–acetic, acid–methanol-water 80:15:12:4) as reported in the method of Bligh and Dyer (1954), modified by Kates (1972) and Card (1973). Menaquinones extraction was done as described by Kröger (1978), followed by HPLC separation using a RP18 column and acetonitrile/isopropanol as mobile phase. For extraction of the whole-cell fatty acids, cells were harvested at the logarithmic phase of growth and investigated according to the standard protocol of Microbial Identification System (Sherlock Version 6.1; MIDI database: TSSA6) (Sasser 1990).

### Bioassay analysis

Antibacterial assays against different Gram-positive and Gram-negative bacteria, fungi and yeast were conducted as previously described (2015). The suspension of test bacteria (OD600 = 0.01) was prepared in Mueller Hinton Broth medium contained (0.5% casein peptone, 0.5% protease peptone, 0.1% meat extract and 0.1% yeast extract, pH 7.0) and suspension of fungi and yeast (OD600 = 0.05) in MYC medium (1.0% glucose, 1.0% phytone peptones and 50-mM HEPES (11.9 g/L), pH 7.0). 20 µl of crude extract was added to the first row horizontally which contained 280 µl of the microbial test suspension and well mixed, 150 µl withdrawn and transferred to the next row wells. This was repeated serially until the end and 150 µl was discarded from the last row in order to obtain a dilution gradient of 66.6, 33.3, 16.6, 8.3, 4.2, 2.1, 1.0 and 0.52 µg/ml. All plates were incubated on a microplate shaker incubator (SB: Heidolph Titramax 1000) for 24 h at 165 rpm at 30 °C (Surup et al. 2015).

## Results and discussion

### Phylogenic analysis

**Fig. 1:**
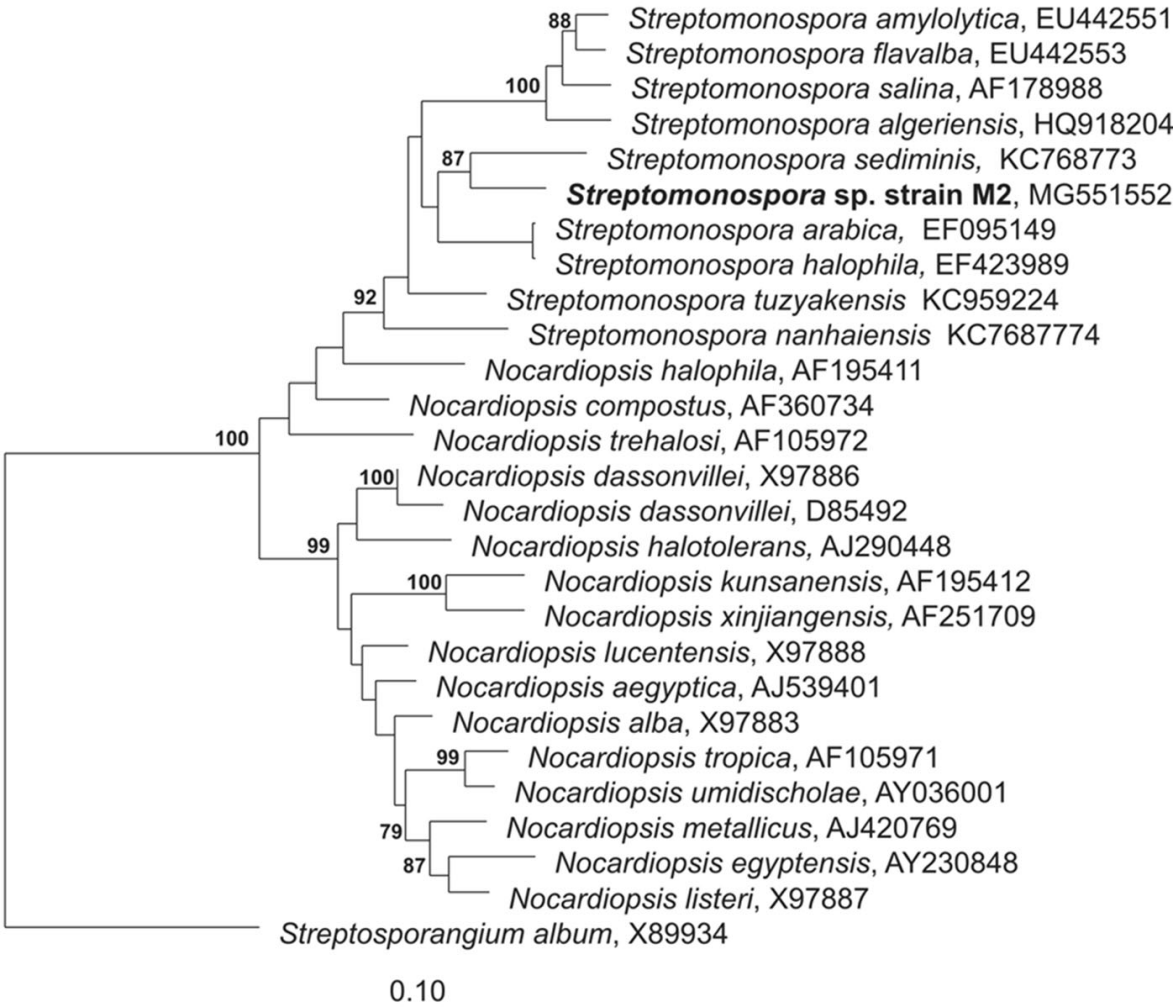
Distance matrix tree (calculated with ARB) showing the position of the strain M2^T^ and type strains of the genus *Streptomonospora* and *Nocardiopsis*. Only bootstrap values > 70% are shown. 1000 calculations

The comparison of the 16S rRNA gene sequence from strain M2^T^ with 16S gene sequences deposited at the EzTaxon database (EzBioCloud.net)  revealed that M2^T^showed closest relationship to strains belonging to the genus *Streptomonospora.* The M2^T^ 16S rRNA gene sequence showed highest similarity to the 16S rRNA gene sequences of strains *S. halophila* DSM 45075^ T^, *S. arabica* DSM 45083^ T^ and *S. sediminis* DSM 45723^ T^ with 98.05, 97.74, and 97.66%, respectively. The phylogenetic tree construction revealed the same three closest type strains to M2^T^, and their relation is shown in the Maximum-likelihood phylogenetic tree (Fig. 1). Moreover, a phylogenomic tree was generated based on the whole-genome sequence of the M2^T^ strain and its closest phylogenetic neighbors by using the Type (Strain) Genome Server (TYGS). The phylogenomic tree revealed closest relationship between M2^T^ and *Streptomonospora alba* YIM 90003^ T^ as well as *Streptomonospora salina* DSM 44593^ T^ (Figure S3). Thus, it can be concluded that strain M2^T^ belongs to the genus *Streptomonospora.* The Sanger 16S rRNA gene sequence was 100% identical to four 16S rRNA gene sequences in the genome of M2^T^ and one differed with 1 nt. M3 is another strain, which has been isolated from the same sand sample. Since strain M3 had identical physiological and chemotaxonomic characteristics, with the exclusion of a divergent Ribotyper pattern (Figure S2) this study focused on the intensive characterisation of strain M2^T^ and the produced thiopeptides.

DNA-DNA hybridisation was performed with genomic DNA from strain M2^T^ and DNA from the three closely related type strains. The level of DDH similarity between M2^T^ and *Streptomonospora halophila* DSM 45075^ T^ was 52.8/45.8%, *Streptomonospora arabica* DSM 45083^ T^ 44.4/39.2% and *Streptomonospora sediminis* DSM 45723^ T^ 21.3/38.9%. In *silico* digital DDH (dDDH) analysis was performed based on the M2^T^ genome sequence. The dDDH analysis identified *Streptomonospora alba* YIM 90003^ T^ as the most closely related type strain. The dDDH value between the two genomes was 26.64% (24.2–29.1%) (using GBDP distance formula 2). The calculated difference of the G + C content between both strains was 0.26%. Thus, it can be inferred that strain M2^T^ represents a novel species within the genus *Streptomonospora*.

The dendrogram based on MALDI-TOF mass spectra showed that the spectra of strain M2^T^ differs from those of *S. halophila* DSM 45075^ T^ and other phylogenetically related actinomycetes (Figure S1). However, the resolution of this method is not sufficient for demonstrating strain-specific differences of the two isolates (Schumann and Maier 2014). The RiboPrinter system has been shown before to have a discriminatory power for differentiation of bacteria at the level of species and even strains (Bruce 1996; Schumann and Pukall 2013). The results obtained by MALDI-TOF MS analysis support the outcome of phylogenetic analyses that M2^T^ is a new species and part of a separate clade within the genus *Streptomonospora.*

### Genome analysis

The final assembly of the whole genome sequence of M2^T^ revealed that the genome consists of two circular contigs with lengths of 5,878,427 bp (chromosome) and a potential prophage/episome with 89,998 bp. The chromosome has a predicted DNA G + C content of 72.1% and includes 5,218 coding sequences, 58 tRNAs, and 15 rRNAs (Table 1). Based on the cluster of orthologous groups (COGs) function categories the distribution of genes is accessible in Table S3.

**Table 1:** Statistical data on the strain M2^T^ genome

Attribute	Value	Percentage%
DNA, total number of bases	5,814,190	100
DNA coding number of bases	4,883,388	83.9
DNA G + C number of bases	4,193,912	72.1
DNA scaffolds	2	100
CRISPR Count	3	
Genes total number	5218	100
Protein coding genes	5107	97.8
Regulatory and miscellaneous features	15	0.2
RNA genes	96	1.8
rRNA genes	15	0.2
5S rRNA	5	0.1
16S rRNA	5	0.1
23S rRNA	5	0.1
tRNA genes	58	1.1
Other RNA genes	23	0.4
Protein coding genes with function prediction	3539	67.8
coding genes without function prediction	1568	30.0
Protein coding genes with enzymes	1087	20.8
Protein coding genes coding signal peptides	203	3.8
Protein coding genes with COGs3	3569	68.4
Protein coding genes with KOGs3	988	18.8
Protein coding genes with Pfam3	3890	74.5
Protein coding genes with TIGRfam3	1122	21.5
Protein coding genes with SMART	1042	19.9
Protein coding genes with SUPERFam	4047	78.0
COG clusters	1679	47.0
Pfam clusters	2026	52.0

### Phenotypical characteristics

**Fig. 2:**
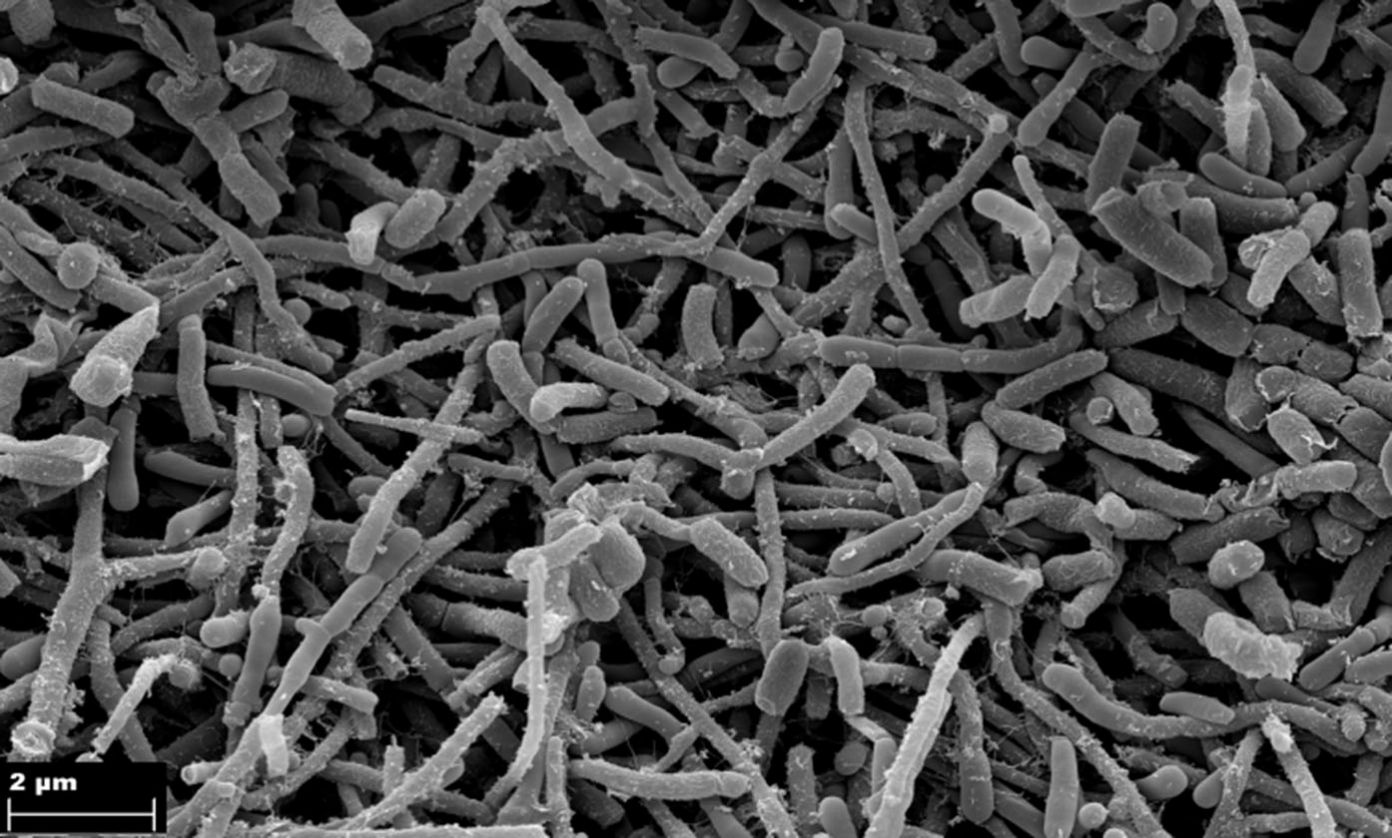
Scanning electron micrographs of strain M2^T^ grown on ISP3 medium for 28 days at 30 °C showing spore chains and single spores. Bars represent 2 μm.

Strain M2^T^ grew on all ISP media with yellow-ivory substrate mycelium and white aerial mycelium. The aerial mycelium was not very abundant. Diffusible pigments were observed on ISP2, ISP3, ISP5, ISP7 and Sutter synthetic media with tyrosine and without tyrosine. All twelve type strains of the genus *Streptomonospora* mentioned in this study produced soluble pigments with lemon yellow-light ivory color under our growth conditions. Before this study, no diffusible pigments have been reported for members of the genus *Streptomonospora*. The general characteristic of strain M2^T^ such as morphology, aerial mycelium, substrate mycelium, production of diffusible pigments, production of melanin pigment, in comparison to the closest related type strains are shown in Table S 2. Strain M2^T^ formed both single spores and spore chains on aerial mycelium but single spores were not determined on substrate mycelium, which was similar to *S. nanhaiensis*, *S. arabica* and *S. halophila*. Straight to flexuous, short spore chains were observed with wrinkled surface and single spores with smooth surface, which were non-motile, oval to cylindric (Fig. 2). The strain utilised glucose, xylose, arabinose, fructose, and rhamnose as carbon sources. M2^T^ grew in media with NaCl concentrations of 0–15% (optimum 7–10% NaCl) at pH 4–9 (optimum pH 7–8) and at temperatures between 20–37 °C (optimum 28–30 °C). Predominant diamino acid was meso-diaminopimelic acid. The cell sugars were ribose, galactose, and glucose. The polar lipids of strain M2^T^ were diphosphatidylglycerol (DPG), phosphatidyl glycerol (PG), phosphatidylinositol (PI), phosphatidylcholine (PC), phosphatidylethanolamine (PE), three glycolipids (GL1-3), two unknown phospholipids (PL1, PL2) and two unknown lipids (Figure S4). The predominant fatty acids (> 10%) of strain M2^T^ were *iso-*C_16_:_0_ (33.1%), *anteiso*-C_17:0_ (24.4%) and 10-methyl C_18_:_0_ (13.6%). The distribution of fatty acids detected for M2^T^ was in accordance with those of members of the genus *Streptomonospora* (Zhang et al. 2013). Quinone analysis revealed MK-10(H_4_), MK-10(H_6_), MK-11(H_6_), and MK-11(H_8_) as predominant quinones. Comparative profiles of cellular features for strains M2^T^, *S. halophila* DSM 45075^ T^*, S. sediminis* DSM 45723^ T^*, S. arabica* DSM 45083^ T^ and *S. salina* DSM 44593^ T^ are listed in Table 2.

**Table 2:** Phenotypic and chemotaxonomic characteristics of strain M2^T^ and closely related type strains of the species of the genus *Streptomonospora*

	1	2	3	4	5
Single spore surface	Smooth	Wrinkled/Smooth	Wrinkled	Smooth/Wrinkled	Wrinkled
NaCl concentration for growth (%, w/v)	0–15	0–20	0–20	0–15	0–20
Optimum NaCl concentration (%, w/v)	7–10	10	0–7	5	15
Growth temperatur (C°)	20–40	20–45	15–45	15–40	nd
Optimum growth temperatur (C°)	28–30	28–30	28	28–30	28
Whole-cell sugars*	Gal, Glu, Man, Rib	Gal, Man Glu, Rib	Gal, Glu, Rib	Glu, Gal	Glu, Gal, Rib, Man
Predominant menaquinones (> 10%) MK**	MK-10 (H_4_), MK-10 (H_6_), MK-10 (H_8_), MK-11 (H_6_)	MK-10 (H_0_), MK-10 (H_2_), MK-10 (H_4_), MK-10 (H_6_), MK-10 (H_8_), MK-11 (H_4_), MK11 (H_6_)	MK-10 (H_6_), MK-11 (H_4,6,8_)	MK-10 (H_4_), MK-10 (H_6_)	MK-10 (H_8_), MK-10 (H_6_), MK-10 (H_4_), MK-10 (H_2_)
Diagnostic phospholipids***	DPG, PG, GL, PE, PL, PI, PC, L	DPG, PG, PC, PI, PL, GL, PE, L	DPG, PC, PG, PI, PIM, PGL, GL, PL	PC, PIM, DPG, PME, PG, PI	PG, PI, PC, 2MPE, PL
Major fatty acid (> 10%)	i-C_16:0_, ai-C_17:0_, 10-methyl C_18:0_	i-C_16:0_, ai-C_16:0_	iC_16:0_, C_16:0_	iso-C_16:0_, 10-methyl C_18:0_, ai-C_17:0_; iso-C_17:0_, iso-C_18:0_, iso-C_16:0_	ai-C_17:0_, i-C_16:0_, 10-methyl C_18:0_
DNA G + C (mol%)	72.1	72.1	70.7	72.3	72.9

Strains: 1, M2^T^; 2, *S*. *halophila* DSM 45075^ T^; 3, *S*. *sediminis* DSM 45723^ T^; 4, *S*. *arabica* DSM 45083^ T^; 5, *S*. *salina* DSM 44593^ T^. Data of strains *S*. *halophila* DSM 45075^ T^ and *S*. *salina* DSM 44593^ T^ are from this study

*Whole-cell sugars: Gal, galactose; Glu, glucose; Man, mannose; Rib, ribose, **MK, menaquinones, ***Diagnosis phospholipids: DPG, diphosphatidylglycerol; PG, phosphatidyl glycerol; PIM, phosphatidylinositol mannosidase; PI, phosphatidyl inositol; PC, phosphatidylcholine; PME, phosphatidyl-N-methyl ethanolamine; PE, phosphatidylethanolamine; GL, glycolipid; PL, phospholipid

The analysis on enzyme activity showed that M2^T^ has no beta-galactosidase activity, which was in contrast to the three analysed *Streptomonospora* type strains. M2^T^ was hydrolysed gelatin and trypsin as observed for *S. sediminis* DSM 45723^ T^ and *S. arabica* DSM 45083^ T^. Cysteine aryl-amidase activity was found to be negative for strain M2^T^. Additional comparative data from API ZYM and API Coryne analysis are listed in Table S1.

### Antibiotic production capacity

Minimal inhibition concentration with ratio (1 µg/ml) of crude extracts (1 mg/ml) from M2^T^ showed high activity against Gram-positive bacteria, such as *Staphylococcus aureus* Newman (0.52 µg/ml), *Micrococcus luteus* DSM 1790^ T^ (0.52 µg/ml), and *Bacillus subtilis* DSM 10^ T^ (0.52 µg/ml). No antibacterial activity was observed against tested Gram-negative bacteria, such as *Escherichia coli* DSM 1116^ T^ and *Pseudomonas aeruginosa* PA14 DSM 19882^ T^, except for *Legionella pneumophila* NCTC 11,192 (12.5 µg/ml)*.* No antibiotic activity was observed against fungal and yeast organisms, such as *Candida albicans* DSM 1665^ T^, *Pichia anomala* DSM 6766^ T^ and *Mucor himalis* DSM 2656^ T^ in this approach.

### Secondary metabolite production profile

**Fig. 3:**
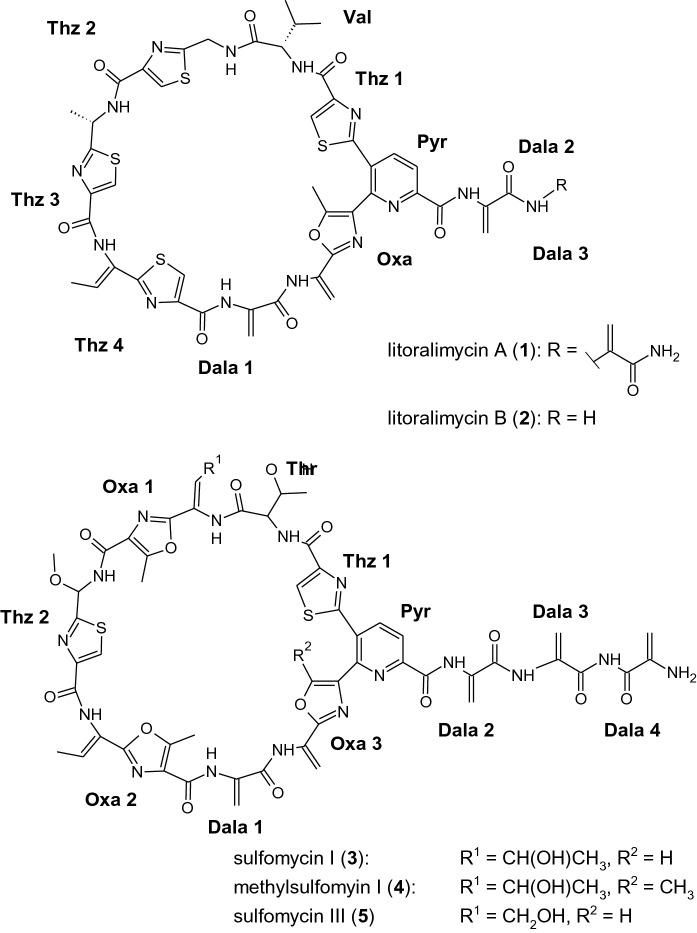
Chemical structures of thiopeptides 1–5 produced by *Streptomonospora* sp. M2^T^

In a previous study, we isolated two new cytotoxic compounds, named litoralimycin A and B from strain M2^T^ (Khodamoradi et al. 2020). However, the production of litoralimycins 1 and 2 could not explain the observed antibacterial properties of crude extracts of strain M2^T^ against the Gram-positive bacteria, since these thiopeptides do not possess antibacterial properties. Therefore, we conducted a bioassay-guided fractionation of the crude extract of M2^T^ and isolated metabolite 3 by preparative HPLC. Its molecular formula C_54_H_52_N_16_O_16_S_2_ was deduced from its quasimolecular ion cluster at *m*/*z* 1245.3260 in the HRESIMS spectrum. ^1^H NMR data identified metabolite 3 as sulfomycin I (Fate et al. 1996). The chemical structures of the metabolites isolated from strain M2^T^ are shown in Fig. 3.

Furthermore, additional active fractions contained congeners 4 and 5 with the molecular formulae C_53_H_50_N_16_O_15_S_2_ and C_53_H_50_N_16_O_16_S_2_, derived from their HRESIMS peaks at *m/z* 1259.3421 and 1231.3104, respectively. These were identified as methylsulfomycin I (Vijaya Kumar et al. 1999) and sulfomycin III (Kohno et al. 1996) according to their ^1^H NMR spectra and HRESIMS data.

Since a strong growth inhibition of Gram-positive bacteria has been described for sulfomycins I–III (Kohno et al. 1996), the robust bioactivity of the M2^T^ crude extract against *Staphylococcus aureus* Newman, *Micrococcus luteus* DSM 1790^ T^, and *Bacillus subtilis* DSM 10^ T^ can be explained by the production of 3–5.

### Encoded secondary metabolite gene clusters

In order to investigate the genetic potential of strain M2^T^ for the production of antibiotics, the genome sequence was analysed using the bioinformatic tool antiSMASH v.5.0 (Blin et al. 2019). AntiSMASH analysis led to the identification twelve predicted secondary metabolite biosynthetic gene clusters (BGCs). Four of the BGCs matched known clusters for isorenieratene (Krügel et al. 1999), geosmin (Jiang et al. 2007), ectoine (Prabhu, 2004), and SapB (Kodani et al. 2004) with 100% similarity. Another two BGCs showed > 80% similarity to clusters encoding fusachelin (Dimiser et al. 2008) and radamycin/globimycin (Kaweewan et al. 2018). The remaining BGCs were predicted to encode three polyketides, one nonribosomal peptide, one lanthipeptide, and one ectoine (Figure S5). As outlined above, M2^T^ has been shown to produce the thiopeptides litoralimycin A and B (Khodamoradi et al. 2020), as well as sulfomycin I, methylsulfomycin and sulfomycin III. Gene cluster region 4 detected by antiSMASH analysis harbors a predicted thiopeptide BGC (BGC4), which is expected to code for the thiopeptide biosynthesis of M2^T^ (Figure S5). BGC4 shows similarity to other known thiopeptide BGCs encoding radamycin/globimycin in S. *globisporus* subsp. *globisporus*, berninamycin A from in *S. bernensis* (Malcolmson et al. 2013) and TP-1161 from a marine *Nocardiopsis* strain with cluster similarity values of 94%, 46% and 33%, respectively (Engelhardt et al. 2010) (Figure S6). BLASTP analysis of individual genes from BGC4 additionally revealed high similarity of the cluster to the recently published sulfomycin I BGC of *S. viridochromogenes* (Du et al. 2020) (Figure S7a). Both BGCs only differ in the thiopeptide precursor encoding gene, whereby one gene (*sulA*) is present in the sulfomycin cluster of *S. viridochromogenes* but two small open reading frames (ORFs) (*lit11, lit12*) are found within BGC4 of M2^T^ (Figure S7, Table S4). *lit11* and *lit12* encode the predicted precursor peptides Lit11 (51-aa) and Lit12 (50-aa), whereby Lit11 harbors a N-terminal 17-aa core peptide (CP) sequence consisting of the amino acid sequence pattern SCTTTGCTTSSSSSSSS and Lit12 a 16-aa CP sequence of SCVGCACTCSSTSSSS. The Lit11 CP sequence is 100% identical to the SulA CP sequence described for sulfomycin biosynthesis in *S. viridochromogenes*, suggesting that *lit11* encodes the sulfomycin I thiopeptide precursor in M2^T^ (Figure S7b). The Lit12 CP is highly similar to the CP sequence of WP_078651956.1 involved in radamycin biosynthesis in *S. globisporus* subsp. *globisporus* with a difference of only two amino acids (Figure S7b). Due to the structural similarity of the litoralimycins with radamycin (Khodamoradi et al. 2020), it can be expected that Lit12 encodes the peptide precursor for litoralimycin biosynthesis in M2^T^.

### Emended description of the genus *Streptomonospora* Cui et al. 2001 emend. Zhang et al. 2013

The description of *Streptomonospora* is as given by Cui et al. (2001), emended by Lie et al. (2003) and by Zhang et al. (2013). With the following amendments, *Streptomonospora* grows on all ISP media and Suter synthetic medium with tyrosine and without tyrosine. Diffusible pigments were demonstrated on most ISP media. Members of the genus can degrade the polysaccharide xyloglucan and arabinan.

## Conclusion

Cultural, morphological, and chemotaxonomic markers besides phylogenomic tree analysis revealed that strain M2^T^ belongs to the genus *Streptomonospora.* The strain could be distinguished from the closely related type strains by several phenotypic characteristics (Table 2). Two different Phospholipids (PLA, PLx) were observed in M2^T^ but not in *Streptomonospora halophila* DSM 45075^ T.^ Some menaquinones including MK-10 (H_0_), MK-10 (H_2_) and MK-11 (H4) were determined in *Streptomonospora halophila* DSM 45075^ T^ but not in strain M2^T^. Cysteine arylamidase activity could not be observed for strain M2^T^, whereas it was positive in all compared three type strains. β-galactosidase activity was detected in all three closest type strains but not in strain M2^T^. Gelatin hydrolysis activity was determined for strains M2^T^, *S. sediminis* DSM 45723^ T^ and *S. arabica* DSM 45083^ T^ but it was negative for *S. halophila* DSM 45075^ T^. Furthermore, A different Ribotyper pattern of strain M2^T^ and closest type strain *S. halophila* DSM 45075^ T^ could specifically distinguish them from each other. In conclusion, based on genotypic and phenotypic features strain M2^T^ represents a novel species of genus *Streptomonospora*, for which the name *Streptomonospora litoralis* sp. nov. is proposed.

### Description of *Streptomonospora litoralis* sp. nov.

*Streptomonospora* litoralis (li.to.ra′lis. L. masc. adj. litoralis of the shore). Gram–positive, aerobic actinomycete. The color of substrate mycelium varied depending on the medium; in ISP2 (honey yellow), ISP3 (white), ISP4 (white), ISP5 (light ivory), ISP6 (golden yellow), ISP7 (ivory), Suter synthetic agar media with/o tyrosine (ochre yellow). Aerial mycelium was detected in all media except of ISP4; the color of aerial mycelia is pure white. Diffusible pigments are produced on ISP2, ISP3, ISP5, and ISP7 also on Suter synthetic media with/o tyrosine. Melanin was not produced on ISP6, ISP7 and SSM with/o tyrosine. At maturity on ISP3 medium, aerial mycelium forms spore chains (wrinkled surface) and single non-motile spores (smooth surface). Growth occurs on ISP2 medium with 0–15% NaCl (w/v), at pH 4–9, in 20–40 °C; optimum of growth determined with 7–10% NaCl, pH 7.0–8.0 at 28 °C. Positive for alkaline phosphatase, esterase (C4), esterase lipase (C8), leucine aryl amidase, valine aryl amidase, acidic phosphatase, naphthol-AS-BI-phosphohydrolase, α-galactosidase, α-glucosidase, glucosidase. Positive in hydrolyses of pachyman, laminarin, rhamnogalacturunan, pectin, xylose, arabinoxylose, hydroxyethyl-cellulose, barley-β-glucan, and gelatin. No hydrolyses of amylose, pullulan, dextran, galactan, rhamnose, galactorunan, α-cellulose, Avicel-cellulose, chitosan, casein, galactose. Predominant menaquinones > 5% include MK-10(H_4_), MK-10(H_6_), MK-10(H_8_), MK-11(H_6_), and MK-11(H_8_). The polar lipids consist of diphosphatidylglycerol (DPG), phosphatidyl glycerol (PG), phosphatidyl inositol (PI), phosphatidylcholine (PC), phosphatidylethanolamine (PE), glycolipid (GL), phospholipid (PL). Crude extract from M2^T^ shows high activity against Gram-positive bacteria, such as *Staphylococcus aureus* Newman (0.52 µg/ml), *Micrococcus luteus* DSM 1790^ T^ (0.52 µg/ml), and *Bacillus subtilis* DSM 10^ T^ (0.52 µg/ml). Strain M2^T^ produces cytotoxic thiopeptides litoralimycin A and B, sulfomycin I, methylsulfomycin I and sulfomycin III.

The type strain is M2^T^ (= DSM 106425^ T^ = NCCB 100650^ T^) isolated from the sand of Cuxhaven beach. The G + C content of the genomic DNA of M2^T^ is 72.1 mol%. The GenBank accession numbers of the 16S rRNA gene and the genome sequence of M2^T^ are MG551552 and CP036455, respectively. The genome comprises a circular 5,878,427 bp large chromosome and a 89,998 bp large potential prophage/episome. Production of thiopeptides litoralimycin A and B (Khodamoradi et al. 2020) and sulfomycin I, III and methylsulfomycin. An additional strain affiliated to that species is M3 (= DSM 107,533).

## Supplementary Information

Below is the link to the electronic supplementary material.

Supplementary file1 (DOCX 904 kb)

## Data Availability

The GenBank accession number for the 16S rRNA gene sequence of strain M2^T^ is MG551552. The GenBank accession number for complete genome of strain M2^T^ is CP036455.
