# Supplementary material for: Streptomonospora litoralis sp. nov., a halophilic thiopeptides producer isolated from sand collected at Cuxhaven beach
Source: Antonie Van Leeuwenhoek. 2021 Aug 6;114(10):1483–96. doi: 10.1007/s10482-021-01609-4 (PMC8448680; doi:10.1007/s10482-021-01609-4)
Supplement: Supplementary file 1 — Supplementary file1 (DOCX 904 kb) [file 10482_2021_1609_MOESM1_ESM.docx]

**Supplementary Information**

***Streptomonospora litoralis sp*. nov., a halophilic thiopeptides producer isolated from sand collected at Cuxhaven beach**

Shadi Khodamoradi *^a^*, Richard L. Hahnke *^b^*, Yvonne Mast ^b^ Peter Schumann *^b^*, Peter Kämpfer ^c^, Michael Steinert *^e^*, Christian Rückert ^f^, Frank Surup ^g^, Manfred Rohde *^d^*, Joachim Wink *^a^*.

^a^ Department of Microbial Strain Collection, Helmholtz Centre for Infection Research, 38124 Braunschweig, Germany

^b^ Leibniz Institut DSMZ-German Collection of Microorganisms and Cell Cultures, 38124 Braunschweig, Germany

^c^ Institut für Angewandte Mikrobiologie, Justus-Liebig-Universität Giessen; Heinrich-Buff-Ring 26–32; D-35392 Giessen, Germany

^d^ Central Facility for Microscopy, Helmholtz Centre for Infection Research, 38124 Braunschweig, Germany

^e^ Institut für Mikrobiologie, Technische Universität Braunschweig, Spielmannstr. 7, 38106 Braunschweig, Germany

^f^ Technology Platform Genomics, Center for Biotechnology, Bielefeld University, D-33615 Bielefeld, Germany

^g^ Microbial Drugs Department, Helmholtz-Centre for Infection Research, 38124 Braunschweig, Germany

**Joachim Wink** is the corresponding author

Email: [Joachim.Wink@helmholtz-hzi.de](mailto:Joachim.Wink@helmholtz-hzi.de)

ORCID iD: <https://orcid.org/0000-0001-9675-0276>

*Streptomonospora tuzyakensis* DSM 45930^T^

*Nocardiopsis xinjiangensis* DSM 44589^T^

*Streptomonospora nanhaiensis* KCTC 29145^T^

*Streptomonospora halotolerans* JCM 30347^T^

*Streptomonospora sediminis* DSM 45723^T^

*Streptomonospora halophila* DSM 45075^T^

*Streptomonospora arabica* DSM 45083^T^

*Streptomonospora salina* DSM 44593^T^

*Streptomonospora flavalba* DSM 45155^T^

*Streptomonospora amylolytica* DSM 45171^T^

*Nocardiopsis algeriensis* DSM 45462^T^

0

100

200

300

400

500

600

700

800

900

1000

Distance Level

***Streptomonospora* M2** DSM 106425^T^

Figure S 1. Dendrogram showing the similarity of MALDI-TOF mass spectra of cell extracts of strains M2^T^, and of closely related type strains of the genera *Streptomonospora* and *Nocardiopsis*.


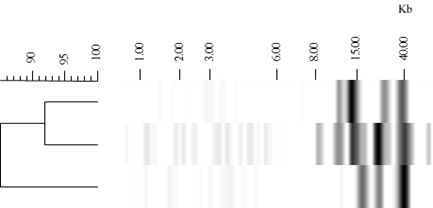


*S.* M2 DSM 106425^T^

*S.* M3 DSM 107533

*S. halophila* DSM 45075 ^T^

Figure S 2. Difference of PvuII RiboPrint patterns of strain M2^T^, strain M3 and the closely related type strain *Streptomonospora halophila* DSM 45075^T^. Cluster analysis was performed by UPGMA based on the Pearson correlation coefficient by using the BioNumerics software (Applied Maths, Version 7.6.1).


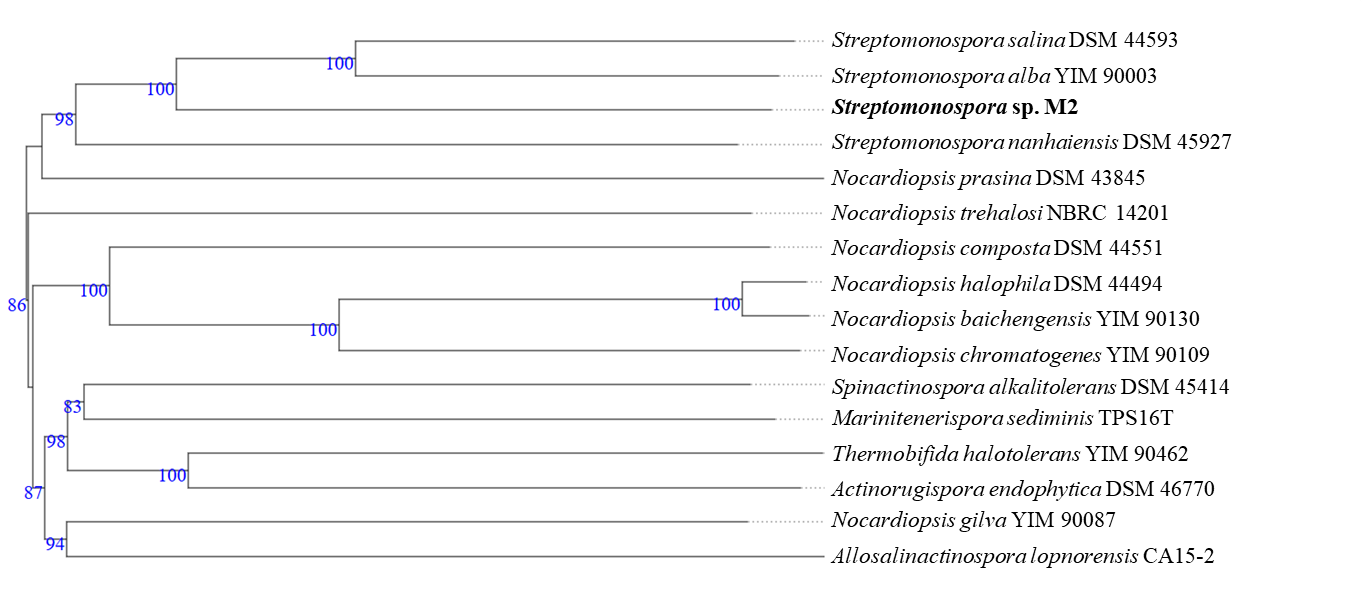


Figure S 3. Whole-genome sequence tree generated with the TYGS web server for strain M2^T^ and closely related species. Tree inferred with FastME from GBDP distances calculated from genome sequences. The branch lengths are scaled in terms of GBDP distance formula d5. The numbers above branches are GBDP pseudo-bootstrap support values > 60% from 100 replications, with an average branch support of 84.4%.


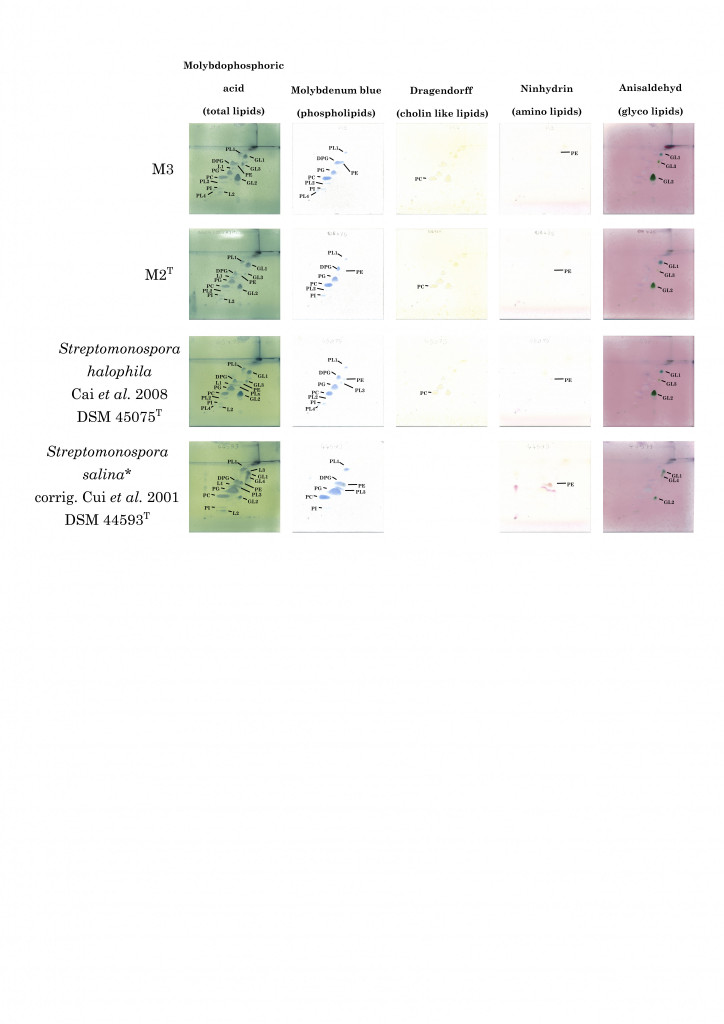


Figure S 4. Polar lipid profiles of strain M2^T^ and close related *S. halophila* DSM45075^T^ and *S. salina* DSM44593^T^ detected on TLC plates by diagnostic spray reagents: molybdophosphoric acid for total lipid-, molybdenum blue for phospholipid-, dragendorff reagent for alkaloids and tertiary amine-, ninhydrin for primary amino acid-, anisaldehyd for glycolipid detection. DPG, diphosphatidylglycerol; PG, phosphatidyl glycerol; PI, phosphatidylinositol; PC, phosphatidylcholine; PE, phosphatidylethanolamine; GLx glycolipid; PLx, phospholipid; Lx, lipid.

Table S 1. Comparative enzyme utilisation analyzed by API ZYM and API Coryne. 1, M2^T^; 2, *S. halophila* DSM 45075^T^; 3, *S. sediminis* DSM 45723^T^; 4, *S. arabica* DSM 45083^T^. Data are from this study, for all strains phosphatases alkaline, esterase lipase (C8), leucine aryl amidase, valine aryl amidase, phosphatase acid, naphthol-AS-BI- phosphohydrolase, phosphatase acid, α-glucosidase, α-galactosidase, β-glucosidase and chymotrypsin are positive while all members showed a negative effect for pyrazinamidase, xylose fermentation, β-glucuronides, mannitol, maltose, ribose fermentation, lactose fermentation, glucose fermentation and sucrose fermentation . + positive; - negative, w weak, v variable.

|  | **1** | **2** | **3** | **4** |
| --- | --- | --- | --- | --- |
| **Api ZYM** |  |  |  |  |
| Lipase (C14) | - | v | - | - |
| Cysteine arylamidase | - | w | + | + |
| Trypsin | w | - | + | + |
| β-galactosidase | - | + | + | + |
| β-glucosidase | + | v | + | + |
| N-acetyl-β-glucose amidase | v | v | + | + |
| α-mannosidase | - | - | - | + |
| **Api Coryne** |  |  |  |  |
| Nitrate reduction | - | - | + | + |
| Pyrrolidonyl arylamidase | - | + | - | - |
| Urease | v | v | + | + |
| Gelatin (hydrolysis) | + | - | + | + |

Table S 2. Culture characteristic of strain M2^T^ in comparison to related type strains of genus *Streptomonospora.* 1, M2^T^*;* 2*, S. halophila* DSM 45075^T^; 3 *S. sediminis* DSM 45723^T^; 4, *S. arabica* DSM 45083^T^; 5, *S. salina* DSM 44593^T^. All data are from this study. –, no pigment

| Medium | characterization | 1 | 2 | 3 | 4 | 5 |
| --- | --- | --- | --- | --- | --- | --- |
|  |  |  |  |  |  |  |
|  | Substrate mycelium | Honey yellow | Honey yellow | Maize yellow | light ivory | Maize yellow |
| ISP2 | Aerial mycelium | Pure white | Signal white | - | - | Pure white |
|  | Soluble pigments | Lemon yellow | - | - | - | Maize yellow |
|  | Substrate mycelium | White | Ivory | Sand yellow | Light ivory | Beige |
| ISP3 | Aerial mycelium | Pure white | Pure white | Pure white | - | Pure white |
|  | Soluble pigments | Ivory | - | Sand yellow | Ivory | Sand yellow |
|  | Substrate mycelium | White | Light ivory | Light ivory | Light ivory | Light ivory |
| ISP4 | Aerial mycelium | - | signal white | - | Pure white | Oyster white |
|  | Soluble pigments | - | - | - | - | Ivory |
|  | Substrate mycelium | Light ivory | Ivory | Ivory | Ivory | Ivory |
| ISP5 | Aerial mycelium | Pure white | Signal white | Pure white | Pure white | Pure white |
|  | Soluble pigments | Light ivory | - | Light ivory | Light ivory | Ivory |
|  | Substrate mycelium | Golden yellow | Maize yellow | Sand yellow | Ivory | Honey yellow |
| ISP 6 | Aerial mycelium | Pure white | - | - | - | Pebble grey |
|  | Soluble pigments | - | - | Sand yellow | - | - |
|  |  |  |  |  |  |  |
|  | Substrate mycelium | Ivory | Golden yellow | Brown yellow | Ivory | Maize yellow |
| ISP7 | Aerial mycelium | Pure white | Cream | Tele grey | Pure white | Pure white |
|  | Soluble pigments | Light ivory | Sand yellow | Beige | Light ivory | Single yellow |
|  | Substrate mycelium | Ochre yellow | Brown beige | Clay Brown | Curry | Brawn beige |
| SSM+T | Aerial mycelium | Pure white | Pure white | Pure white | Pure white | Pure white |
|  | Soluble pigments | Light ivory | Ivory | Olive brown | Ivory | Sand yellow |
|  | Substrate mycelium | Ochre yellow | Olive yellow | Green beige | Curry | Curry |
| SSM-T | Aerial mycelium | Pure white | Signal white | Pure white | Pure white | Pure white |
|  | Soluble pigments | Light ivory | - | Ivory | Light ivory | Light ivory |

Table S 3. Number of genes associated with functional categories in COG.

| Function | Code | Value | Percentage% |
| --- | --- | --- | --- |
|  |  |  |  |
| Amino acid transport and metabolism | [E] | 331 | 7.6 |
| Carbohydrate transport and metabolism | [G] | 337 | 7.8 |
| Cell cycle control, cell division, chromosome partitioning | [D] | 47 | 1.0 |
| Cell motility | [N] | 13 | 0.3 |
| Cell wall/membrane/envelope biogenesis | [M] | 178 | 4.1 |
| Chromatin structure and dynamics | [B] | 1 | 0.0 |
| Coenzyme transport and metabolism | [H] | 242 | 5.6 |
| Cytoskeleton | [Z] | 1 | 0.0 |
| Defense mechanisms | [V] | 131 | 3.0 |
| Energy production and conversion | [C] | 248 | 5.7 |
| Extracellular structures | [W] | 12 | 0.2 |
| Function unknown | [S] | 206 | 4.7 |
| General function prediction only | [R] | 537 | 12.4 |
| Inorganic ion transport and metabolism | [P] | 219 | 5.0 |
| Intracellular trafficking, secretion, and vesicular transport | [U] | 36 | 0.8 |
| Lipid transport and metabolism | [I] | 226 | 5.2 |
| Mobilome: prophages, transposons | [X] | 45 | 1.0 |
| Nucleotide transport and metabolism | [F] | 88 | 2.0 |
| Posttranslational modification, protein turnover, chaperones | [O] | 159 | 3.6 |
|  |  |  |  |
| RNA processing and modification | [A] | 1 | 0.0 |
| Replication, recombination and repair | [L] | 123 | 2.6 |
| Secondary metabolites biosynthesis, transport and catabolism |  |  |  |
| Signal transduction mechanisms | [Q] | 215 | 4.9 |
| Transcription | [T] | 229 | 5.3 |
| Translation, ribosomal structure and biogenesis | [K] | 447 | 11.0 |
|  | [J] | 206 | 4.7 |
|  |  |  |  |
| Not in COG | [NA] | 1649 | 31.6 |


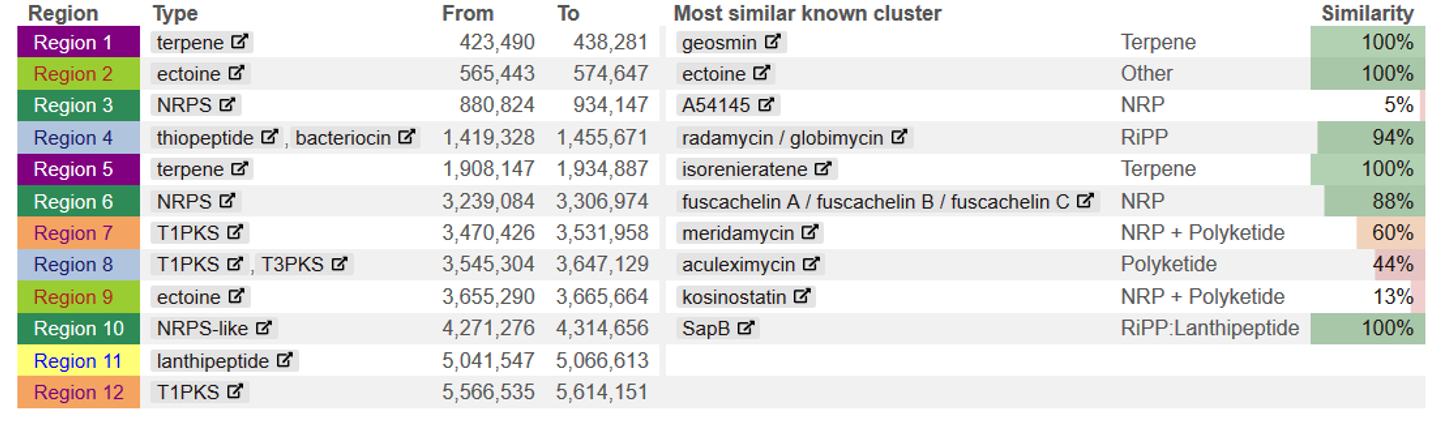


Figure S 5. List of predicted secondary metabolite gene clusters for strain M2^T^ identified by analysis of the M2^T^ genome sequence with the bioinformatic tool antiSMASH 5.0.


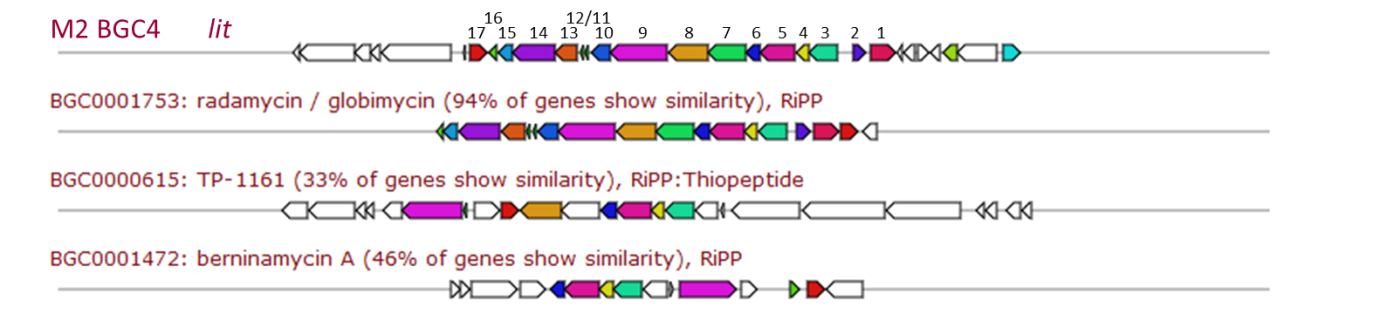


Figure S 6. BGC4 from strain M2^T^ in comparison to other known thiopeptide gene clusters. The predicted M2^T^ biosynthetic genes are numbered as listed in Table S1. Homologous genes are indicated by the same arrow color. RiPP = Ribosomally synthesized and post-translationally modified peptide.


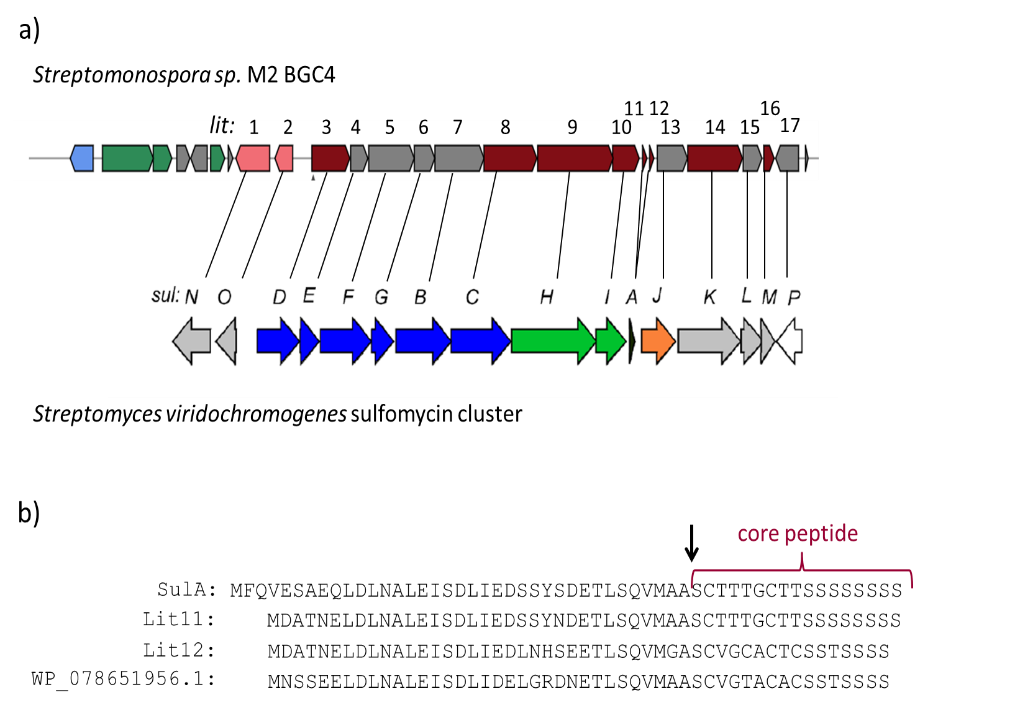


Figure S 7 a) Schematic presentation of BGC4 from strain M2^T^ (upper part) and the sulfomycin cluster from *Streptomyces viridochromogenes* b) Alignment of amino acid sequences from different precursor peptides from thiopeptide producers. SulA: sulfomycin producer *S. viridochromogenes*; Lit11/12: litoralimycin producer *S*. M2^T^, WP_078651956.1: globimycin/radamycin producer *S. globisporus subsp*. *globisporus*. Red brace indicates core peptide sequence; black arrow indicated prepeptide cleavage position.

Table S 4. Strain M2^T^ Region 4 genes and their deduced functions. ORF, open reading frame; †ID/SM, % identity/similarity of amino acid sequences. *S. alba, Streptomonospora alba; S. auratus, Streptomyces auratus; S. incarnatus, Streptomyces incarnatus; S.* sp. PA3, *Streptomonospora* sp. PA3; *S. virido., Streptomyces viridochromogenes* ATCC 29776*; M. nebraskense, Mycobacterium nebraskense; N. gilva, Nocardiopsis gilva; N. halotolerans, Nocardiopsis halotolerans; N. sinuspersic, Nocardiopsis sinuspersic.* (-) means no homologs present

| **Gene-No.** | **ORF** | **Predicted function** | **ID/SM**† | **Matched strain** | **Accession number** | **Sulfomycin-related homolog** | **ID/SM**† | **Matched strain** | **Accession number** |
| --- | --- | --- | --- | --- | --- | --- | --- | --- | --- |
| 6160 | *orf1* | ABC transporter ATP-binding protein | 83/87 | *S. alba* | WP_040270763 | - | - | - | - |
| 6155 | *orf2* | two-component sensor histidine kinase | 62/70 | *S.* sp. PA3 | WP_156002281 | - | - | - | - |
| 6150 | *orf3* | response regulator transcription factor | 84/90 | *S. alba* | WP_040270764 | - | - | - | - |
| 6145 | *orf4* | hypothetical protein | 61/73 | *S. alba* | WP_040270745 | - | - | - | - |
| 6140 | *orf5* | hypothetical protein | 70/81 | *N. halotolerans* | WP_017571558 | - | - | - | - |
| 6135 | *orf6* | MarR family transcriptional regulator | 77/84 | *N. sinuspersic* | WP_077690797 | - | - | - | - |
| 6130 | *orf7* | RidA family protein | 87/94 | *N. gilva* | WP_051060844 | - | - | - | - |
| 6125 | *lit1* | cytochrome P450 | 88/93 | *S. auratus* | WP_040899041 | SulN | 87/93 | *S. virido.* | QJC58222 |
| 6120 | *lit2* | class I SAM-dependent methyltransferase | 81/89 | *S. auratus* | WP_106430513 | SulO | 81/90 | *S. virido.* | QJC58216 |
| 6115 | *lit3* | YcaO-like family protein | 84/90 | *S. auratus* | WP_040899039 | SuD | 84/90 | *S. virido.* | QJC58217 |
| 6110 | *lit4* | cyclodehydratase | 84/89 | *S. auratus* | WP_006605028 | SulE | 83/89 | *S. virido.* | QJC58220 |
| 6105 | *lit5* | TpaE | 82/89 | *S. incarnatus* | AKJ11409 | SulF | 82/89 | *S. virido.* | QJC58219 |
| 6100 | *lit6* | TpaF | 80/86 | *S. incarnatus* | AKJ11410 | SulG | 81/86 | *S. virido.* | QJC58218 |
| 6095 | *lit7* | hypothetical protein | 73/82 | *S. auratus* | WP_144044220 | SulB | 72/80 | *S. virido.* | QJC58223 |
| 6090 | *lit8* | YcaO-like family protein | 82/86 | *S. auratus* | WP_051007002 | SulC | 80/87 | *S. virido.* | QJC58224 |
| 6085 | *lit9* | lantibiotic dehydratase | 86/91 | *S. auratus* | WP_106430410 | SulH | 85/91 | *S. virido.* | QJC58225 |
| 6080 | *lit10* | hypothetical protein | 81/87 | *S. auratus* | WP_078568713 | SulI | 79/85 | *S. virido.* | QJC58226 |
| 6075 | *lit11* | thiocillin/thiostrepton family thiazolyl peptide | 82/91 | *S. auratus* | WP_051006902 | SulA | 81/94 | *S. virido.* | QJC58227 |
| 6070 | *lit12* | thiocillin/thiostrepton family thiazolyl peptide | 76/85 | *S. auratus* | WP_051006902 | SulA | 68/88 | *S. virido.* | QJC58227 |
| 6065 | *lit13* | hypothetical protein | 57/88 | *S. incarnatus* | AKJ11416 | SulJ | 75/87 | *S. virido.* | QJC58228 |
| 6060 | *lit14* | radical SAM protein | 91/95 | *S. incarnatus* | AKJ11417 | SulK | 91/95 | *S. virido.* | QJC58229 |
| 6055 | *lit15* | hypothetical protein | 82/89 | *S. incarnatus* | AKJ11418 | SulL | 80/89 | *S. virido.* | QJC58230 |
| 6050 | *lit16* | hypothetical protein | 90/95 | *S. auratus* | WP_006605017 | SulM | 88/94 | *S. virido.* | QJC58231 |
| 6045 | *lit17* | 23S rRNA methyltransferase | 83/90 | *S. incarnatus* | AKJ11419 | SulP | 83/89 | *S. virido* | QJC58232 |
| 6040 | *orf8* | IS481 family transposase | 77/87 | *M. nebraskense* | WP_047324341 | - | - |  | - |
